# What Amphibians Can Teach Us About the Evolution of Parental Care

**DOI:** 10.1146/annurev-ecolsys-102221-050519

**Published:** 2023-08-04

**Authors:** Eva Ringler, Bibiana Rojas, Jennifer L. Stynoski, Lisa M. Schulte

**Affiliations:** 1 Division of Behavioural Ecology, Institute of Ecology and Evolution, University of Bern, Bern, Switzerland; 2 Department of Interdisciplinary Life Sciences, Konrad Lorenz Institute of Ethology, University of Veterinary Medicine Vienna, Vienna, Austria; 3 Department of Biology and Environmental Science, University of Jyväskylä, Jyväskylä, Finland; 4 Clodomiro Picado Institute, University of Costa Rica, San José, Costa Rica; 5 Faculty of Biological Sciences, Goethe University Frankfurt, Frankfurt am Main, Germany

**Keywords:** fertilization mode, terrestriality, protection, transport, nutrition, parental sex roles

## Abstract

Parenting is considered a key evolutionary innovation that contributed to the diversification and expansion of vertebrates. However, we know little about how such diversity evolved. Amphibians are an ideal group in which to identify the ecological factors that have facilitated or constrained the evolution of different forms of parental care. Among, but also within, the three amphibian orders—Anura, Caudata, and Gymnophiona—there is a high level of variation in habitat use, fertilization mode, mating systems, and parental sex roles. Recent work using broad phylogenetic, experimental, and physiological approaches has helped to uncover the factors that have selected for the evolution of care and transitions between different forms of parenting. Here, we highlight the exceptional diversity of amphibian parental care, emphasize the unique opportunities this group offers for addressing key questions about the evolution of parenting, and give insights into promising novel directions of research.

## 1 Introduction

The diversity of ways in which animals have evolved to improve the survival of their offspring is astounding (Royle et al. 2012). That is particularly true among the amphibians, which include a father frog that slurps up his eggs and avoids digestion for weeks until his then froglets hop out from his vocal sac, a mother caecilian that encourages her young to chew off her lipid-filled skin as essential nutrition, and an aquatic salamander that carefully curls her fresh eggs into leaf rolls to avoid losing them to hungry fish (mentioned in Schulte et al. 2020). As the first land-dwelling tetrapods, amphibians may have arrived at these and dozens of other parental innovations in response to selection pressures to sustain their fish-like young in semipermeable and externally fertilized eggs while adapting to terrestrial habitats (Furness et al. 2022, Vági et al. 2019).

Each year, herpetologists and behavioral ecologists uncover fascinating new forms and functions of parental care among the three amphibian orders (over 7,500 species of anurans, approximately 800 species of urodeles, and over 200 species of gymnophiones) (Frost 2023), adding depth and detail to our overall understanding of parental care evolution in this vertebrate clade (Schulte et al. 2020). Such research is particularly timely and relevant because amphibians are the class of vertebrates most threatened by anthropogenic change on local and global scales, with 41% of over 8,500 extant species experiencing population declines (IUCN 2022). Thus, more work to describe and synthesize the evolutionary novelties, mechanisms, and ecological contexts of diverse forms of amphibian parental care is urgently needed.

Our aims in this review are to provide a current synthesis of the behavioral, morphological, and physiological adaptations of amphibians performing parental care. We highlight the unique selective pressures that may have contributed to the scale of diversity in parental investment across this group and to the high level of convergent evolution seen in certain forms of parental care. Following an introductory framework describing parental care and its features among the amphibians, we break down the three principal functions of care in this vertebrate group—protection, transport, and nutrition. We then integrate the observed forms and functions in terms of their shared mechanisms and consequences, with the ultimate objective of identifying key goals and hypotheses for future research in this field.

## 2 What is Parental Care, and Why Should We Care about It?

Parental care can be defined as nongametic contributions by parents that are likely to increase the survival and growth of offspring, sometimes referring only to parental behaviors and sometimes to a broader definition that also includes physiological and morphological inputs to offspring (Royle et al. 2012). While some authors reporting on parental care may specify that they refer only to parental care after fertilization or after hatching or birth (Crump 1996), other authors take into consideration all offspring life stages, including very early stages such as nest building. In this review, we refer to parental care with respect to all inputs and stages. A similar term, parental investment, specifically refers to the reduction of a parent’s future reproductive success as a result of the investment that the parent makes to increase the survival of current offspring, in the currency of fitness (Trivers 1972). This term, along with parental effort (fitness costs due to care of all offspring), parental effect (influence of the parent’s phenotype on the phenotype of their young), and parental expenditure (time, energy, and resources spent on offspring) have unfortunately been used inconsistently and interchangeably in the literature.

Why should we study parental care? In short, parental care plays a central role in the evolution of animals, as it is directly linked to life history strategies, mating systems, sexual selection, sociality, cooperation, and conflict (Balshine 2012, Royle et al. 2012). As parenting can impose considerable costs on the caregiver in terms of energy expenditure, survival likelihood, and reduced future reproductive success, parents are expected to optimize their behavioral responses in such a way that the offspring’s survival is ensured at minimal parental effort (Trivers 1972). At the same time, each offspring is expected to demand more than parents are willing to invest (termed parent–offspring conflict) (Trivers 1974). Conflicts of interest occur not only between offspring and parents but also between the parents themselves (Houston et al. 2005). Because time devoted to parental care reduces the time available for courtship and mating, the evolution of parental care is closely linked to sexual selection (Royle et al. 2012, Trivers 1972). Moreover, in several vertebrate taxa, parental care is constrained by physiological abilities, such as the inability of most males to produce nutritive substances, such as milk and eggs (see Section 4.3). Consequently, the relative costs and benefits of providing particular forms, intensities, or durations of care are likely to vary significantly between mothers, fathers, and offspring, and therefore, evolutionary conflicts are likely to arise.

Most animals do little more for offspring than provide gametes, perhaps some yolk, and a suitable environment for the early developmental period. Among the subset of animals that do provide care, taxonomic groups differ quite considerably with regards to the extent and duration of care, as well as whether mothers, fathers, or both parents provide care. Among endothermic vertebrates (birds and mammals), parental care is the predominant reproductive strategy. In birds, care is primarily biparental and includes nest building, thermal brooding, and food provisioning, whereas in mammals, care is primarily maternal and includes gestation, lactation, and physical protection. Moreover, in mammals, parenting evolved at the base of their lineage, is associated with considerable morphological and physiological adaptations to the female body (i.e., structures to support lactation after giving birth), and is therefore considered homologous (Balshine 2012). Parental care is less common among ectotherms such as fish, amphibians, reptiles, and invertebrates (Balshine 2012, Schulte et al. 2020). In these groups, it has evolved independently multiple times, presumably in response to the challenges imposed by unfavorable or unpredictable environments in terms of humidity, water availability, and ambient temperature. Most of the research on parental care has focused on birds and mammals, although this strong taxonomic bias (Stahlschmidt 2011) appears to be diminishing slightly with time as more reports are made available from other taxa with diverse parental care modes such as invertebrates, fish, reptiles, and amphibians.

## 3 Evolutionary Routes to Parenting in Amphibians

### 3.1 Invasion of Terrestrial Habitats

**Figure 1:**
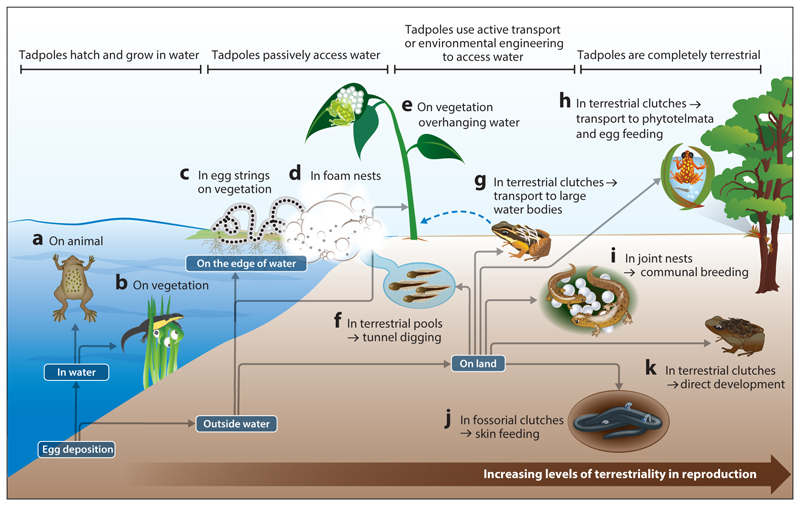
Evolutionary transitions from aquatic to terrestrial reproduction in amphibians are associated with diverse behavioral, physiological, and morphological adaptations. This figure shows examples of the evolution of parenting in response to increased terrestriality of adult amphibians. Several species have retained their aquatic reproductive mode, e.g., (*a*) fertilized eggs submerged in specialized brood pouches and (*b*) eggs wrapped in vegetation under water. Some species deposit their eggs directly adjacent to water bodies, into which tadpoles drop after hatching, e.g., (*c*) egg strings on vegetation just above the water surface, (*d*) foam nests at the edge of water, and (*e*) eggs on vegetation hanging over water. Sometimes clutches are deposited farther from water bodies, which requires active involvement of the parent to provide tadpoles with access to water, e.g., (*f*) terrestrial pools, from which parents dig tunnels to connect them with bigger water bodies, and (*g*) terrestrial clutches with tadpole transport to large water bodies. In some species, reproduction occurs in the complete absence of large water bodies, e.g., (*h*) terrestrial clutches with tadpole transport to small water bodies and subsequent egg feeding by the mother, (*i*) communal breeding in joint nests, (*j*) fossorial offspring and intermittent skin feeding by the mother, and (*k*) direct development within eggs.

As a trait inherited from their fully aquatic sarcopterygian ancestors, amphibian eggs lack shells (i.e., amnion), which makes them highly susceptible to environmental threats. Therefore, most amphibians are still highly dependent on water for reproduction, which has led to the evolution of alternative ways to keep eggs and larvae hydrated, even while outside of water. Early amphibians likely faced this challenge via a rapid diversification of reproductive modes, which allowed them to deposit their eggs on the edge of, above, or independent from water. This diversity in reproductive modes has been, and remains, a trademark of amphibians (Crump 2015, Luz Nunes-de-Almeida et al. 2021). A general shift toward reproduction in terrestrial habitats has likely evolved as a response to intense predation on aquatic eggs and larvae (Alford 1999, Magnusson & Hero 1991), although predation also constitutes a major threat in nonaquatic environments (Warkentin 1995, Warkentin et al. 2005). In addition, intense intrasexual selection likely has favored terrestrial reproduction due to a reduction in simultaneous polyandry and the associated risks of sperm competition in nonaquatic environments (Zamudio et al. 2016). Especially in tropical environments, which are not only hotspots of biodiversity in terms of species numbers but also with respect to reproductive strategies (Hödl 1990), we see the full spectrum from aquatic to terrestrial reproduction. Given that terrestrial environments are hostile for anamniotic eggs due to the high risks of desiccation (Touchon & Worley 2015), physical damage, predation, and exposure to diseases (Martin & Carter 2013), evolutionary transitions toward terrestrial reproduction required considerable behavioral, physiological, and morphological adaptations (Zamudio et al. 2016). Nonetheless, the benefits of invading nonaquatic habitats to occupy novel ecological niches likely exceeded the costs of adapting to terrestrial reproduction, and parenting often played a key role in facilitating this transition (Figure 1). The selective pressures shared across terrestrial habitats likely promoted the convergent evolution of parental care in different clades.

Parenting is considered one of the key traits linked to the expansion of amphibians into nonaquatic niches (Furness et al. 2022), especially in species with external fertilization. Diverse parental modes have evolved to increase offspring survival by overcoming the threats imposed by terrestrial environments, for example, egg brooding by physical contact and urination to keep eggs hydrated and reduce fungal infections, removal of unviable eggs to avoid the spread of infection, and active defense from predators (e.g., Crump 1996, Delia et al. 2013, Poo & Bickford 2013; see also Section 4.1). Recent phylogenetic comparative analyses suggest that the durations of both care and protection of offspring by males and females have coevolved with terrestrial reproduction (Furness et al. 2022, Vági et al. 2019). Counterintuitively, although parenting is seen as a mechanism to protect offspring from dangers associated with harsh environmental conditions (Martin & Carter 2013), amphibian species with terrestrial oviposition occur exclusively in regions with high annual precipitation such as humid tropical and subtropical environments (Gomez-Mestre et al. 2012, Haddad & Prado 2005, Vági et al. 2022). Habitats with high precipitation throughout the year likely provide favorable conditions for a large number of reproductive modes (Crump 2015, Hödl 1990), whereas habitats with low precipitation and seasonality mainly support aquatic eggs and larvae (Silva et al. 2012). Thus, it seems that certain climatic prerequisites, especially in terms of humidity, needed to be fulfilled before parenting could become adaptive, as parents are also vulnerable to overheating and desiccation (Vági et al. 2020, 2022).

### 3.2 The Tadpole Dilemma

**Figure 2:**
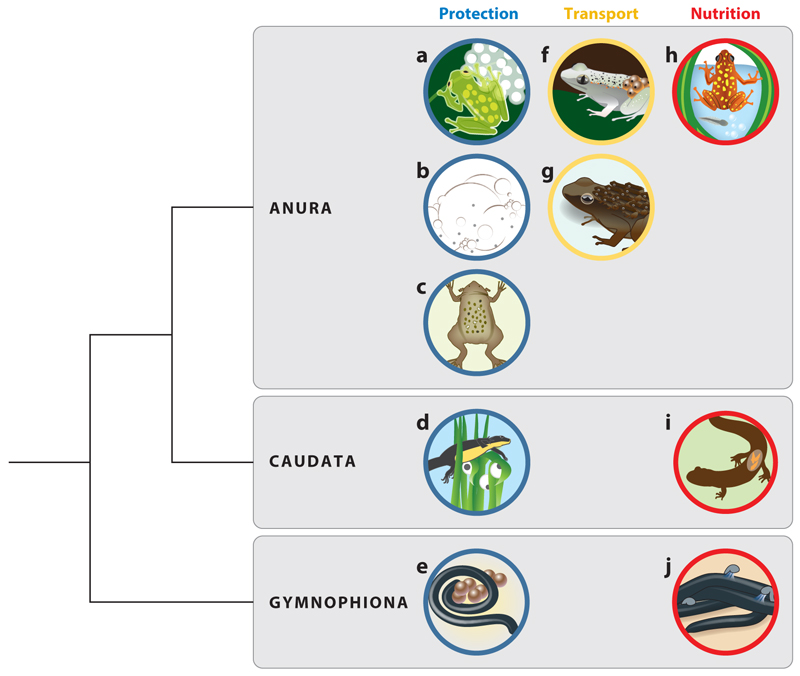
Convergent evolution of parenting across amphibians. The three major parenting modalities—protection (*blue circles*), transport (*yellow circles*), and nutrition (*red circles*)—have evolved multiple times independently both across and within taxonomic lineages. Depicted examples highlight different strategies for protection: (*a*) sitting on/next to clutch, (*b*) foam nests, (*c*) eggs developing in specialized body structures, (*d*) wrapping of single eggs in vegetation, (*e*) coiling around eggs; transport: (*f*) of eggs, (*g*) of larvae or juveniles; nutrition: (*h*) egg feeding, (*i*) uterine feeding, (*j*) skin feeding.

The transition to terrestrial reproduction imposed considerable challenges not only on eggs that were deposited partially or fully outside of water but also on the next developmental stage: the larvae. It is a major dilemma that amphibian larvae from terrestrial clutches still have gills and need water to survive and complete their development. To overcome this fundamental problem, amphibians have evolved an impressive set of strategies (Figure 1). On one hand, certain reproductive modes have evolved to entirely circumvent an aquatic tadpole stage, such as direct development of larvae inside eggs (Gomez-Mestre et al. 2012) or viviparity (Velo-Antón et al. 2007). On the other hand, the evolution of behavioral strategies, such as the deposition of eggs on vegetation hanging over water into which tadpoles drop after hatching (Delia et al. 2017, Warkentin 1995), ecological engineering to allow larvae to commute to larger water bodies (Kaminsky et al. 1999), or the active transport of tadpoles to water bodies by parents themselves (Fouilloux et al. 2021), guarantee that the newly hatched larvae reach the aquatic environment they require to complete their development. These diverse solutions have evolved independently in several amphibian clades, representing striking examples of convergent evolution (Figure 2). One case of stunning radiation of care modes within a very small clade is the genus *Anomaloglossus* from the Guiana shield, where exotrophy, endotrophy, and transportation in various forms (including direct development during transport) have evolved within a comparatively small biogeographic region and over relatively short evolutionary time (Vacher et al. 2017).

### 3.3 Egg Versus Clutch Size

Parental care in amphibians tends to be associated with larger eggs and smaller clutches (Summers et al. 2006). However, recent research indicates that this association is probably mediated by a common underlying factor, namely terrestrial reproduction (Furness et al. 2022, Vági et al. 2019). In general terms, the transition from aquatic to terrestrial egg laying was accompanied by changes in key life history traits such as an increase in egg size and a reduction in clutch size (Gomez-Mestre et al. 2012), which reflects the trade-off between water balance and oxygen uptake of terrestrial eggs. Large eggs have a better volume-to-surface ratio than small eggs, which increases their water holding capacity but decreases oxygen diffusion. Also, the tight packaging of eggs in terrestrial clutches and their physical properties, such as thick jelly layers and additional membranes, are considered adaptations to prevent dehydration, but these same adaptations hinder oxygen diffusion (Seymour & Bradford 1995, Warkentin et al. 2005). Some species have therefore evolved reduced protective layers to raise oxygen supply (Altig & McDiarmid 2007), which in turn may have selected for parental care in terms of protection and brooding. However, comparative studies that provide a broad view on this relationship are lacking. The subsequent transition in vertebrate evolution to amniote eggs (i.e., eggs with external shells) was a major step that totally decoupled reproduction from water and set the stage for the evolution of reptiles and the radiation of birds and mammals (Sumida & Martin 1997).

### 3.4 Diversity of Parental Care Among Amphibia

The majority of amphibians do not provide any care to their offspring. In most species, eggs are deposited by females directly into large water bodies, where they are externally fertilized by males, and embryos develop without any further involvement by either parent (Wells 2007). Only approximately 10–20% of extant species are classified as being parental (Balshine 2012, Schulte et al. 2020). Interestingly, these few species are not limited to one taxonomic unit, but rather, we find convergent evolution of similar parental strategies—including various morphological, physiological, and behavioral adaptations—in all three orders of amphibians (Anura, Caudata, and Gymnophiona). Over 30 forms of parental care are known in amphibians, and they fall along a wide spectrum of complexity, from more basic forms such as egg attendance and nest building to strikingly intricate forms such as brooding of embryos in distinct parts of the body, transporting larvae dorsally to water pools, and larval feeding with unfertilized eggs (Schulte et al. 2020). However, the prevalence, duration, and also complexity of care are very different across and within these three taxonomic groups (Schulte et al. 2020), which makes amphibians excellent models to investigate the ecological and social factors that have facilitated—or constrained—the evolution of parenting.

In anurans (frogs and toads), only 10–20% of species provide parental care (the actual percentage depends on the definition of care), but the subset of species that have evolved some form of parenting do so with an astounding diversity and complexity (Schulte et al. 2020, Wells 2007). Anurans have the greatest number of described parental care modes (28) among all amphibians (Crump 2015, Schulte et al. 2020), ranging from egg attendance to various ways of transporting and protecting offspring to providing nutrition. In Caudata (newts and salamanders), around 20% of species are currently known to exhibit parental care (Vági et al. 2022), but the variation and also complexity of care is generally lower (8 care modes, with the predominant mode being egg attendance) compared to anurans. Nonetheless, the duration of care is still highly variable in this group, not only between clades but also between species and between sexes within species (Vági et al. 2022). With regard to caecilians (Gymnophiona), we unfortunately know very little about their reproductive strategies, including parental care. This is mainly due to their highly secretive fossorial lifestyle, which makes observational and experimental approaches more challenging compared to the terrestrial, arboreal, and aquatic species in the other two orders (but see Section 4.3). As a consequence, only three parental modes have been described so far for caecilians (Schulte et al. 2020). Given the big gap in knowledge about general life history traits and behavioral strategies in this group, we presume that the prevalence of parental care in caecilians is severely underestimated.

### 3.5 Who Cares?

Amphibians are unique among the vertebrates in that they exhibit both paternal and maternal care in similar proportions, and biparental care has also evolved in various lineages (Furness & Capellini 2019). This variation makes amphibians particularly well suited for studying the factors that select for sex-specific involvement in care. When we look at parental roles in amphibians—and also across vertebrates in general—fertilization mode seems to be the main predictor of whether the male or the female remains with the brood and provides care (Furness & Capellini 2019, Vági et al. 2022). While female-only care has evolved mainly in internal fertilizers, male-only care occurs almost exclusively in externally fertilizing species, such as anurans and ancestral urodeles with aquatic reproduction, where it has evolved multiple times from no care (Furness & Capellini 2019). With external fertilization, males may benefit from staying with the clutch to ensure fertilization, giving the female the opportunity to desert the clutch immediately after oviposition and leave any further parental care to the male. From comparative work in fishes, we know that the certainty of paternity, which is much higher in external than in internal fertilizing species, is an important factor that predicts investment in care by males (Neff 2003). Behavioral strategies to ensure high levels of paternity, such as egg attendance and territoriality to thwart sneaker males and clutch piracy (Hettyey & Pearman 2003, Vieites et al. 2004), probably have further promoted male-only care in species with external fertilization.

In contrast, maternal care is mainly found in urodeles with internal fertilization or in species in which parental care is physiologically constrained to females (i.e., egg feeding, see Section 4.3). In urodeles, internal fertilization may have facilitated the occupation of terrestrial environments and subsequently selected for further maternal involvement in offspring care, thereby allowing them to become fully independent from water for reproduction (Vági et al. 2022). In this context, viviparity—the retention of embryos in the maternal body until larval development is completed—can be seen as the most extreme form of maternal care, because it is expected to evolve exclusively from internal fertilization (Furness & Capellini 2019) and requires several anatomical and physiological modifications in both the developing offspring and the maternal reproductive tract.

Biparental care is not common in any of the three amphibian orders, although we see some flexibility regarding parental roles in a few species (Bourne 1998, Ringler et al. 2015). On one hand, social structures such as stable pair bonds and social monogamy, which are typically associated with biparental care in other taxa (Royle et al. 2012), are exceptionally rare in anurans (Brown et al. 2010, Tumulty et al. 2014) and unknown in urodeles and caecilians. On the other hand, in insects, birds, and fishes, biparental care was shown to evolve mainly from female-only care (i.e., males join an already caring female) (Balshine 2012). A recent study involving hundreds of amphibian species suggests that biparental care in amphibians evolves at approximately equal rates from either male-only or female-only care. Once evolved, biparental care shows high rates of transition back toward male or female uniparental care, suggesting that it is an evolutionarily unstable condition (Furness & Capellini 2019).

Similar to biparental care, alloparenting—care provided by unrelated adults—is even less common in amphibians. Mutualistic alloparenting, such as communal breeding as an adaptive strategy, has been reported in the northern two-lined salamander: Multiple females breed in synchrony in a joint nest and benefit from taking turns in providing care to the entire nest (LeGros 2011). From observational studies and clutch foster experiments in anurans, we know that parents might provide care to unrelated eggs or larvae (Cabeza-Alfaro et al. 2021, Ringler et al. 2016, Rodrigues et al. 2011, Valencia-Aguilar et al. 2021). However, these studies describe cases of misdirected alloparenting and do not provide evidence of an adaptive strategy under natural conditions.

## 4 Functions of Parental Care in Amphibians

The threats that were imposed on eggs and larvae when early amphibians shifted from aquatic to terrestrial reproduction then spurred the evolution of diverse reproductive modes. Within some of these modes, additional parental strategies to ensure and enhance offspring survival have evolved. Here, we discuss three principal parental modalities found in amphibians: (*a*) protection of the offspring from biotic and abiotic threats, (*b*) temporary or long-term transport of the offspring, and (*c*) provisioning of nutrition to the offspring.

### 4.1 Protection

Parental care is presumed to evolve in response to biotic and abiotic threats to offspring survival. Of the highly diverse parental care modes found in amphibians, egg attendance (also known as egg guarding and egg brooding) (Delia et al. 2020) is one of the simplest and most widespread care behaviors, which, as a product of convergent evolution, can be found in many species in all three amphibian orders (Crump 2015, Schulte et al. 2020). While in caecilians, egg attendance is performed exclusively by females (Kupfer et al. 2016), in urodeles and anurans, females, males, or even both sexes can be involved (Schulte & Summers 2021, Wells 2007). The proposed reasons why amphibians attend their eggs are numerous and depend on the clutch location and ecological conditions, but the most commonly suggested function is protection from desiccation, pathogens, and predators. In aquatic environments, lack of oxygen and high egg predation pressure are the main risks to developing embryos (Okada et al. 2015, Wells 2007). In most amphibian species, however, egg attendance has evolved in association with terrestrial egg deposition (see Section 3.2). Oviposition on land entails new threats to the eggs that parental caregivers must adapt to, including dehydration, terrestrial predators, and pathogens.

One of these threats, dehydration, is addressed by many species through regular hydration of their terrestrial egg clutches. This form of egg attendance, or brooding behavior, consists of the parent covering the eggs with their body to reduce water loss and/or directly releasing liquid to the eggs through the venter or from their cloaca (Crump 1996). In some lungless salamanders (Plethodontidae), unattended eggs lose more water per hour than eggs attended by a female (Forester 1984). Also, in glass frogs (Centrolenidae), egg attendance positively affects hydration, and parents adjust the frequency of attendance to both weather conditions and egg dehydration status (Delia et al. 2013). Another form of protection, also called egg guarding behavior, is directed at terrestrial predators. It can be either passive or active; for example, female rhacophorid frogs either passively shield their eggs with their body or actively attack egg predators (Poo et al. 2016). Similarly, European lungless salamanders actively bite cannibalistic conspecifics to defend their eggs and also passively coil around their eggs when predators approach, even much larger predators such as rats (Oneto et al. 2010). In poison frogs, eggs might even be cannibalized by male or female conspecifics if not attended by parents (Ringler et al. 2017, Spring et al. 2019). A third major threat to amphibian eggs is pathogens, such as fungal infections (Green 1999). Parental protection can be supplied by removing infected eggs or by preventing fungal growth via vertical transmission of antifungal cutaneous bacteria to the clutch (Lauer et al. 2007). In eleutherodactylid frogs, for example, egg mortality due to fungal infections is sharply reduced when parents regularly sit on their clutches (Bourne 1998).

Although egg attendance is the most widespread form of protection in amphibians, some species provide care by ensuring a safe environment for tadpoles or metamorphs. In some leptodactylid frogs, females stay with their tadpoles after they hatch in shallow waters and actively defend them against predators (Rodrigues et al. 2011). Also, female European lungless salamanders remain with their hatchlings for several weeks at the nesting site (Oneto et al. 2010). Some amphibian parents take steps to protect their offspring from future dangers even before or during oviposition (i.e., prezygotic care, such as nest building). In anurans, for example, parents of numerous species from seven different families build so-called froth, foam, or bubble nests for their eggs, which are produced by whipping, scooping, or blowing air into mucus. In urodeles, several aquatic breeding newts roll their eggs in leaves, which effectively prevents predation (Oriazola & Braña 2003). These nest-building strategies lead to similar solutions through convergent evolution: the protection of the offspring, both before and after hatching, from different biotic and abiotic threats, including desiccation and predation (Gould 2021). Alternatively, parents can protect offspring via embryo retention (e.g., viviparity as the most extreme form), or by transporting eggs, tadpoles, or juveniles in or on their body.

### 4.2 Transport

While many amphibian species deposit clutches terrestrially and stay to attend to the eggs, several other anuran lineages have independently evolved ways to take the offspring along with them (Wells 2007). Two main functions of offspring transport have evolved in different clades. First, long-term carrying of offspring on or inside the parent’s body serves as another form of protection from biotic and abiotic threats. Second, during short-term transportation, parents can release their offspring at the most favorable sites.

Female frogs of the family Hemiphractidae transport their large eggs either exposed on the dorsum or incubated in partially or fully enclosed dorsal brood pouches (so-called marsupial frogs) (del Pino 2018). When eggs are brooded on or under the mother’s skin, the likelihood of egg predation is reduced to cases of direct predation on the female, which can perform a variety of self-defense behaviors (Lourenço-de-Moraes et al. 2016). Furthermore, persistent tissue contact, especially within fully developed brood pouches, drastically reduces the probability of egg desiccation. However, a consequence of brooding embryos within such skin pouches is that parents must maintain specialized structures for respiratory gas exchange (for a review, see del Pino 2018). This special connection between the parent and their eggs can also serve a secondary function: the transfer of nutrients to embryos (Warne & Catenazzi 2016; see also Section 4.3).

Clutch-carrying has evolved independently several times in frogs, with some lineages having direct-developing eggs and others having eggs that hatch into tadpoles and are transported to water bodies (Wells 2007). In species with free-swimming tadpole stages, regardless of whether they hatched on the parent or from a terrestrial clutch, parents must make choices to release the tadpoles in the most preferable aquatic environments (Buxton & Sperry 2016, Fouilloux et al. 2021). Tadpole transport is especially well studied in Neotropical poison frogs (Dendrobatidae and Aromobatidae). In these species, 1–40 tadpoles are carried by either the male or the female to aquatic sites ranging from streams to miniature pools in leaf axils (Weygoldt 1987). The trade-off between pool persistence, food availability, and predator presence, which are all mediated by pool size, is considered a key factor in the evolution of diverse parenting strategies in this frog family (Brown et al. 2010). Large water bodies are generally associated with low risk of desiccation and offer plenty of nutrients to developing tadpoles. Therefore, species that make use of large water bodies usually do not provide any additional care after larval deposition. However, along with increasing pool size, predation risk also increases, which may have selected for the use of gradually smaller water bodies in other dendrobatoid species. Very small water bodies, such as bromeliad axils, indeed offer environments with much lower risk of predation but in turn lack sufficient nutrients for developing tadpoles. As a consequence, maternal provisioning with (usually) unfertilized eggs has evolved in species that use bromeliad axils as pools (Dugas et al. 2016, Schulte et al. 2016, Stynoski 2009, Summers & McKeon 2004; see also Section 4.3).

Before tadpole release, many poison frog species carefully evaluate pools for both biotic and abiotic costs and benefits (e.g., water levels, pH, predators or conspecifics) (Brown et al. 2008, Fouilloux et al. 2021, Rojas 2014, Schulte et al. 2011; see also Section 3). In some dendrobatoids, transporting parents seek to evenly distribute their offspring across a variety of pools to increase the probability of offspring survival (Erich et al. 2015)—a bet-hedging strategy that can also be found in tadpole-transporting discoglossid frogs (Goyes Vallejos et al. 2019). In some cases, tadpole transport extends into tadpole brooding, for example, in myobatrachid hip-pocket frogs, whose tadpoles climb into special brood pouches near the hip and later emerge as fully developed froglets (Ingram et al. 1975). In Darwin’s frogs (Rhinodermatidae), males pick up newly hatched tadpoles, brood and nourish them in their vocal sacs (Goicoechea et al. 1986), and later release them as either late-stage tadpoles or froglets. In some egg-carrying species with direct development, froglets continue to grow on the mother’s dorsum while benefiting from further protection (Jungfer & Böhme 1991). Transport of froglets can also result in even dispersal of the offspring, thereby reducing sibling competition and inbreeding (Bickford 2004). Furthermore, parents can transport juveniles from habitats that are most favorable for eggs (e.g., caves) to habitats more favorable for froglets (e.g., forests) (Diesel et al. 1995). Parents can also improve developmental conditions during transport; male midwife toads (Alytidae) wrap strings of their eggs around their legs, and shift their thermal preferences in favor of their offspring (Lange et al. 2022).

For one transport strategy, however, more research has become impossible: Both species of gastric brooding frogs (*Rheobatrachus*, Myobatrachidae), in which females swallowed their eggs and brooded the offspring in their stomachs, are now extinct due to anthropogenic influences (for further details, see Schulte et al. 2020). Sadly, whether these frogs also fed offspring through stomach tissues and the mechanisms by which they ceased their own gastric function while brooding can no longer be investigated.

### 4.3 Nutrition

Most amphibian species provide only yolk to offspring, which nourishes the embryo until hatching. However, nutritive provisioning—beyond vitellogenic yolk deposition—is common in caecilians (7 of 10 families) (Schulte et al. 2020). In contrast, offspring feeding is rare among frogs (9 of 56 families) and unknown for urodeles, with the exception of a few viviparous members of the family of true salamanders (Salamandridae) (Guex & Greven 1994, Schulte et al. 2020). Species that provide nutrition to offspring tend to have fewer eggs per clutch (Kupfer et al. 2016, Vági et al. 2019), presumably because they are investing resources to produce higher quality offspring. Besides yolk, parental amphibians can provision nutrients via access to food resources for exotrophic offspring, adult tissues to matriphagic or patriphagic offspring, or unfertilized eggs to oophagic offspring. Unfortunately, one of the few known species with patriphagic offspring (*Ecnomiohyla rabborum*) is already extinct.

Parental amphibians may provide indirect access to food resources by choosing favorable sites for egg or larval deposition (Caspers et al. 2015, Fouilloux et al. 2021, Goyes Vallejos et al. 2019). Also, there is some evidence that the proteins in bubble nests can provide nutritional benefits to offspring after hatching (Kusano et al. 2006) and that tunnel-digging frogs may enable tadpole access to additional feeding areas (Kaminsky et al. 1999).

Most endotrophic offspring (i.e., direct developers and nidicolous larvae) receive only yolk from parents, and the eggs and yolk of these species tend to be larger than eggs of species that receive nutrition besides the yolk (del Pino 2018, Wake & Hanken 1996). However, labeled isotope analysis has shown that some endotrophic amphibians, such as marsupial frogs, also receive maternal nutrients that are transferred to the eggs through the brood pouch (Warne & Catenazzi 2016). Similarly, tracers have been used to demonstrate the transfer of nutrients from Darwin’s frog fathers to larval offspring during development in the vocal sac (Goicoechea et al. 1986). Skin feeding, or dermatotrophy, is a common form of provisioning in several caecilian families (Kupfer et al. 2016), which resembles the feeding of offspring with lipid-rich modified epithelial tissue, comparable to the milk of mammals or the crop milk of some birds (pigeons, flamingos, emperor penguins) (Ding et al. 2020). However, it is still unknown if caecilian skin, which tends to be paler than skin of noncaring adults, also provides antibodies, nutrients, and microbiota, as do other vertebrate milks (Jared et al. 2019), and whether similar hormonal processes (i.e., prolactin) modulate its production. Dermatotrophic species generally have oviparous direct-developing larvae with distinctive preadult teeth that they use to peel off maternal skin (Jared et al. 2019, Kupfer et al. 2016). These species also supply their young with a cloacal fluid, but its function and nutritional value are unknown. Dermatotrophy is thought to be an ancestral form of parental care that facilitated the evolution of uterotrophy in caecilians (Wilkinson et al. 2008). Uterotrophic caecilian offspring are viviparous and also have distinctive preadult teeth, which are used to scrape off not skin but rather hypertrophied epithelia in the maternal oviduct (Wake & Dickie 1998). Uterotrophy also occurs in the subspecies of some viviparous salamandrids, which transfer nutrients via epitheliophagy of specialized oviductal tissues (Guex & Greven 1994).

Egg feeding is a parental behavior that has been widely studied in dendrobatid frogs (Brooks et al. 2023, Schulte 2013, Stynoski 2009, Villanueva et al. 2022) but also has independently evolved in other anuran families (Schulte et al. 2020). Strategic egg feeding probably evolved from a scenario in which females repeatedly deposited eggs in the same locations across consecutive mating events, and older siblings exploited and consumed deposited eggs. The adaptive benefits of such high quality food then probably gave rise to more autonomous forms of maternal egg provisioning in several anuran lineages (see also Section 5.1). In the majority of these lineages, egg feeding is facultative, and tadpoles also feed on other items such as detritus, algae, and arthropods. A few species are obligately oophagous, as their tadpoles do not survive or reach metamorphosis without a regular supply of maternal nutritive eggs (Brust 1993, Liang et al. 2002). The neural and hormonal mechanisms by which mother frogs regulate the timing and quantity of costly egg feeding behaviors and ensure that their own offspring are the targets of egg feeding have been the topic of much study in recent years (Fischer et al. 2019c, Schulte & Summers 2017; see also Section 5.3).

Anuran evolutionary transitions between strategies for offspring transport, brooding, and nutrition are dynamic, and often serve multiple functions. In this context, the special case of viviparity can be seen as an adaptation that serves all those three parental modalities: protection, transport, and nutrition (Figure 2).

## 5 Mechanisms and Consequences

None of the three modes of care mentioned in Section 4 occurs in isolation from other aspects of the parents’ behavior, physiology, and life history. Parents are expected to incur costs while providing offspring with care. One such cost is an increased predation risk. Given the length of time that parents must stay in the same spot during egg brooding, or the long distances some need to cover to deposit their tadpoles in adequate rearing sites, it is not surprising that antipredator strategies and parental care behavior have coevolved, at least in some cases.

### 5.1 Coloration

Recent comparative studies on anurans have investigated the link between coloration and parental care and found interesting patterns. For example, among species that provide parental care (375 out of 988 included in the study), most lack sexual dichromatism, regardless of the caregiving sex and regardless of whether the modality of care involves the parent moving or remaining stationary (Seshadri & Thaker 2022). Interestingly, in species with male-only care, males tend to exhibit dorsal stripes, which the authors hypothesize helps them become camouflaged (Seshadri & Thaker 2022). An increased role of crypsis also seems to be an essential factor for glass frog parents (Barnett et al. 2020), which can attend clutches up to 24 hours a day. These frogs can conceal their red blood cells in the liver during periods of sleep, which improves transparency during highly vulnerable periods (Taboada et al. 2022).

In contrast, among poison frogs, aposematic species (i.e., those in which conspicuous coloration is coupled with the possession of secondary defenses such as skin toxins) tend to deposit their newly hatched tadpoles in phytotelmata more frequently than camouflaged, undefended species (Carvajal-Castro et al. 2021). This diversification of breeding site use is thought to be possible due to the extra freedom that aposematism provides, allowing organisms to wander around exploiting resources that less protected (i.e., camouflaged) species cannot (Speed et al. 2010). In the case of poison frogs, aposematism seems to have evolved before phytotelm breeding. Aposematic species also tend to carry fewer tadpoles than camouflaged species (Carvajal-Castro et al. 2021), which may, at least in part, be explained by the fact that the tadpoles carried on the dorsum of a parent may hamper the efficacy of its aposematic signal, rendering it more vulnerable to predator attacks (Toro-Gómez et al. 2022). Moreover, female care occurs predominantly among aposematic phytotelm breeders (Carvajal-Castro et al. 2021), which, in addition, tend to take caring one step further by providing unfertilized eggs to their offspring (Brown et al. 2010). Remarkably, these eggs provide not only food but also chemical defenses (i.e., alkaloids) (Brooks et al. 2023, Stynoski et al. 2014b), which have been shown to be sequestered by tadpoles as soon as granular glands develop (Stynoski & O’Connell 2017) and to protect them from multiple types of predators (Stynoski et al. 2014a). Such defense provisioning occurs, however, only in obligate egg feeders and not in facultative ones (Villanueva et al. 2022; see also Section 4.3). All of these studies indicate that, for species with parental care, it is particularly vital to be either highly camouflaged or, alternatively, to communicate strong toxicity via conspicuous warning colors to potential predators. Unfortunately, these questions have not been addressed in either salamanders or caecilians.

Another interesting link between coloration and parental care has been recently highlighted by an experiment that determined that poison frog tadpoles imprint on the color of the foster parent during tadpole transport (Yang et al. 2019). After maturation, cross-fostered females preferred to court mates of the same color as their foster mother, while cross-fostered males were more aggressive toward rivals that had the color of their foster mother. This finding suggests that rival and sexual imprinting during parental care can reduce gene flow between individuals that bear divergent mating traits, which might even set the stage for speciation (Yang et al. 2019).

### 5.2 Energetics and Locomotor Performance

Besides the external selection pressure imposed by predation, caring parents must cope with internal pressures, too. Caring duties may involve high energetic demands and negative effects on locomotor performance, particularly in the case of parents that transport their offspring over long distances and time (Cuestas Carrillo et al. 2022, Lange et al. 2021, Townsend 1986). Here, again, poison frogs are a good example of how parental care and other traits have evolved in tandem. These frogs have exceptionally high metabolic rates compared to other amphibians (Santos & Cannatella 2011), which may have evolved alongside their specialist diet and, in turn, may have allowed them to travel in search of distant—and sometimes surprisingly vertically high—tadpole deposition sites (Summers 2019). Tadpole transport requires not only high levels of endurance from the parent but also a successful mechanism of tadpole (or egg, in the case of other clades) attachment, so as to prevent them from falling off. While it is known for many egg-carrying species, the mechanism by which tadpoles remain attached to carrying parents is still an unresolved question.

### 5.3 Underlying Mechanisms

Research in the past few years has considerably increased our knowledge of the consequences of parental care for tadpole development and survival as well as the costs incurred by parents. Yet, approaches aimed at unveiling the mechanistic basis of parental care in amphibians are still scarce. Some recent work has shed light on the hormonal and neurobiological mechanisms involved in larval transport and egg provisioning and highlights interesting convergences with other vertebrates. For example, particular hormones and neuropeptides known to mediate parental care behaviors in other vertebrate taxa are also relevant during tadpole transport in male and female poison frogs (Fischer et al. 2019b). Furthermore, while frogs are feeding their tadpoles with unfertilized eggs, the brain regions that are activated (lateral septum and preoptic area) are similar to those activated during nursing behavior in mammals (Fischer et al. 2019c). Interestingly, while hormones and gene expression patterns in the brain are similar between active and observing parents, the levels of neural activity are clearly associated with the performance of caring behavior, regardless of the sex of the caregiver (Fischer & O’Connell 2020). These studies highlight the great potential of amphibians for research on the mechanistic basis of parental sex roles.

## 6 Future Directions in the Study of Amphibian Parental Care

Despite its slow start relative to taxa such as mammals and birds, research on amphibian parental care has been getting traction in recent years, although this progress is still heavily biased toward Neotropical poison frogs (families Aromobatidae and Dendrobatidae) (Schulte et al. 2020). In fact, the rapid surge of work on the correlates, function, and evolution of parental care in this group of frogs is a testament to the fundamental role of natural history studies in our understanding of evolutionary processes. Therefore, we advocate for more research on natural history, as that is the ultimate source of new hypotheses (Endler 2015) and the spark needed to ignite exciting projects. This is particularly true for caecilians, newts, and salamanders, about which we know much less than we do about frogs and toads. Given the current trends of biodiversity loss, which look particularly bleak for amphibians (IUCN 2022), populations, and even species, currently disappear before we even get the chance to study their parental behavior and associated morphological and physiological adaptations (e.g., gastric-breeding frogs) (see Section 4.2). Only after a solid natural history background is available can we move forward with designing ecologically relevant experiments and carrying out comprehensive comparative analyses—as shown by studies on poison frogs. While correlational studies can give a useful overview, it is only through confirmatory studies involving the manipulation of the presence or absence of parents, the hormonal profile, the environmental characteristics, or the availability and form of critical resources, to name a few, that we can really find the answers to long-standing questions about the costs and benefits of parenting.

Phylogenetic comparative analyses have proven to be an excellent approach with which to investigate the conditions that gave rise to certain types of care and the other traits with which these have coevolved (e.g., Carvajal-Castro et al. 2021, Furness & Capellini 2019, Kupfer et al. 2016, Seshadri & Thaker 2022, Vági et al. 2019). The possibilities are endless in this respect, as they are with mathematical models investigating in detail, for example, the costs that each sex performing parental care suffers under specific scenarios or the potential effects of particular anthropogenic threats on the occurrence, intensity, frequency, or duration of parental care. Amphibians offer a high diversity of reproductive modes, ecology, habitats, parental roles, and parental adaptations both within and across taxonomic units, and thus a unique framework with which to answer broad questions about the evolutionary origins of parenting.

Broad background knowledge of the species’ natural history is also a prerequisite for the integration of multiple disciplines, which provides a better understanding of the interplay between behavior, physiology, and ecology in the evolution of parental care. Here, recent technological advances can open up entirely new avenues to link parental care with individual reproductive success, assess cognitive processes linked to parenting, and identify the neuroendocrinological basis for parenting and its role in the evolution of parental sex roles. For example, molecular genetics enable the reconstruction of entire pedigrees (Ringler et al. 2018), miniaturized tracking devices allow for a fine-scale mapping of movement patterns in parenting frogs (Pašukonis et al. 2022), transcriptomic profiling can be used to identify neuronal activity in the brain (Fischer et al. 2019b,c), and CRISPR/Cas9 might allow the manipulation of distinct gene expression patterns that might inhibit or promote parental behaviors [as suggested by Fischer et al. (2021)]. Moreover, novel omics approaches and analytical tools to identify the transfer of microbiota, toxins, chemicals, or nutrients from parents to offspring (see Hakala et al. 2022) will provide new insights into the advantages of parenting for offspring survival and development.

Another reason that amphibians are a great system in which to study the genomic, neuronal, and hormonal mechanisms that underlie parenting is because care in this group has evolved with a wide spectrum of parental sex roles within and among clades, including many cases in which one or the other sex was not physiologically constrained or affected by pair bonding (cf. Fischer et al. 2019a). In some cases, these parental behaviors can be elicited spontaneously. For example, cross-fostering manipulations in poison frogs have shown that the placement of unrelated tadpoles on adult frogs can override the decision-making rules that direct tadpole pickup and can provoke tadpole transport to distant deposition sites by males and females (Pašukonis et al. 2017). Further studies in this and other poison frogs have highlighted the prominent role of spatial memory and learning for tadpole-transporting frogs (Beck et al. 2017, Liu et al. 2019, Pašukonis et al. 2016). Building on profound life-history knowledge, future studies should combine behavioral, physiological, and environmental manipulations to identify which mechanisms are most relevant in ecological natural settings.

Paradoxically, mutualistic alloparental care is a common strategy in fishes, birds, and mammals, whereas it is almost absent in amphibians and probably also in reptiles (Royle et al. 2012). Future studies should investigate amphibians that form aggregations to probe for parental behaviors that might allow for the evolution of alloparenting.

In addition, given the importance of amphibians as ecological indicators of environmental disturbance, more studies should investigate the effects of global change on the different forms of care (Schlippe Justicia et al. 2022). While it has been previously reported that hostile environments do not seem to be associated with any specific type of care (Vági et al. 2020), current trends of climate change and habitat destruction may yet render this finding obsolete. Therefore, both modelling and experimental approaches could be useful to assess whether and how global change impacts the evolution of different forms of parental care (the emergence or loss thereof). Under challenging conditions, one could expect that parents might opt for reduced frequency or intensity of parental care, in anticipation of future mating opportunities under better circumstances (Winkler 1987). Alternatively, parents could increase the frequency or intensity of care in an attempt to maximize the survival probabilities of their offspring, obviously at the expense of their own survival or future opportunities to reproduce.

## 7 Conclusions

Amphibians have evolved an impressive set of strategies to protect their offspring against dehydration, predation, starvation, and various infections and diseases. Across amphibians, we see a strong trend to shift reproduction from aquatic to terrestrial environments, which led to a rapid diversification of reproductive modes in early amphibians. The benefits of occupying novel ecological niches likely exceeded the costs of adapting to terrestrial reproduction—and parenting often played a key role in facilitating this transition. Extant amphibians are characterized by a large variation in life history traits, ecological niches, reproductive modes, and parental sex roles. As such, they represent ideal model systems for studying the origins and diversification of parental care, as well as the social and environmental predictors of parenting.
